# Microbial analysis of in situ biofilm formation in drinking water distribution systems: implications for monitoring and control of drinking water quality

**DOI:** 10.1007/s00253-015-7155-3

**Published:** 2015-12-05

**Authors:** Isabel Douterelo, M. Jackson, C. Solomon, J. Boxall

**Affiliations:** Pennine Water Group, Department of Civil and Structural Engineering, Mappin Street, University of Sheffield, Sheffield, S1 3JD UK; Wessex Water, Claverton Down Rd, Bath, Somerset BA2 7WW UK

**Keywords:** Bacteria, Biofilms, Drinking water distribution systems, Fungi

## Abstract

Biofilm formation in drinking water distribution systems (DWDS) is influenced by the source water, the supply infrastructure and the operation of the system. A holistic approach was used to advance knowledge on the development of mixed species biofilms in situ, by using biofilm sampling devices installed in chlorinated networks. Key physico-chemical parameters and conventional microbial indicators for drinking water quality were analysed. Biofilm coverage on pipes was evaluated by scanning electron microscopy (SEM) and confocal laser scanning microscopy (CLSM). The microbial community structure, bacteria and fungi, of water and biofilms was assessed using pyrosequencing. Conventional wisdom leads to an expectation for less microbial diversity in groundwater supplied systems. However, the analysis of bulk water showed higher microbial diversity in groundwater site samples compared with the surface water site. Conversely, higher diversity and richness were detected in biofilms from the surface water site. The average biofilm coverage was similar among sites. Disinfection residual and other key variables were similar between the two sites, other than nitrates, alkalinity and the hydraulic conditions which were extremely low at the groundwater site. Thus, the unexpected result of an exceptionally low diversity with few dominant genera (*Pseudomonas* and *Basidiobolus*) in groundwater biofilm samples, despite the more diverse community in the bulk water, is attributed to the low-flow hydraulic conditions. This finding evidences that the local environmental conditions are shaping biofilm formation, composition and amount, and hence managing these is critical for the best operation of DWDS to safeguard water quality.

**Electronic supplementary material:**

The online version of this article (doi:10.1007/s00253-015-7155-3) contains supplementary material, which is available to authorized users.

## Introduction

Water utilities invest time and money towards controlling the presence of undesirable microorganisms in drinking water systems. However, the lack of realistic knowledge on the microbiology of drinking water distribution systems (DWDS) and on the involvement of biofilms in processes that deteriorate the performance of the water infrastructure makes existing management strategies unsustainable in the long term. Several aspects of the microbial ecology of DWDS remain unknown and/or the information we have is limited and/or not applicable to real-world conditions. Most of the existent monitoring methods (e.g. faecal indicators) for drinking water are based on detecting microorganisms in bulk water samples from taps and using culture-dependent methods. However, bulk water samples are not representative of attached communities, and given that biofilms represent more than 95 % of the biomass in DWDS (Flemming et al. 2002). monitoring based solely on bulk water samples is a serious oversight. Additionally, it is recognised that culture methods underestimate the actual diversity of microbes in the environment (Amann et al. 1995). The safety and quality of drinking water can be compromised by opportunistic pathogens that can survive within biofilms attached to pipes (Lehtola et al. 2007). Additionally, biofilms play a central role in water discolouration (Husband et al. 2008). changes in water taste and odour (Szewzyk et al. 2000). corrosion, scale formation and even pipe blockage (McNeill and Edwards, 2001).

The difficulty of accessing the internal surface of pipes within operational networks makes the study of biofilms challenging. Most of the information available about DWDS biofilms has been gathered using small-scale laboratory reactors and/or assessing few selected microorganisms under controlled conditions which do not represent the dynamics of diverse communities within real networks (Douterelo et al*.*2014a). Evidence from studies under representative conditions is needed to determine how environmental factors control the formation of biofilms in DWDS and hence their impact on water quality. This research utilised sampling devices which allow for monitoring of biofilm development in situ without disrupting the boundary hydraulic conditions in the pipes (Deines et al. 2010). The devices allowed for analysing physical and compositional characteristics of biofilms which were selected on the basis of their potential applicability in future monitoring campaigns. While bacteria have been widely studied in DWDS, little, if any, attention has been paid to fungi, despite their association with the production of toxins and changes in water taste and odour (Hageskal et al. 2007; Siqueira et al. 2012). Fungi have been detected in drinking bulk water samples using culturing techniques (Doggett, 2000; Gonçalves et al. 2006; Hageskal et al. 2006). This study is a step forward in the research of fungi in DWDS exploring the role of these microorganisms in the development of biofilms by using high-throughput sequencing techniques.

The holistic approach used here, combining water physico-chemical analysis, flow cytometry, microscopy analysis and pyrosequencing, was designed to address knowledge gaps regarding the formation of biofilms in situ in real DWDS*.* This study offers valuable insights on the microbial ecology of DWDS and informs future monitoring strategies to optimise the management of drinking water systems.

## Materials and methods

### Characteristics of sampling sites

**Fig. 1:**
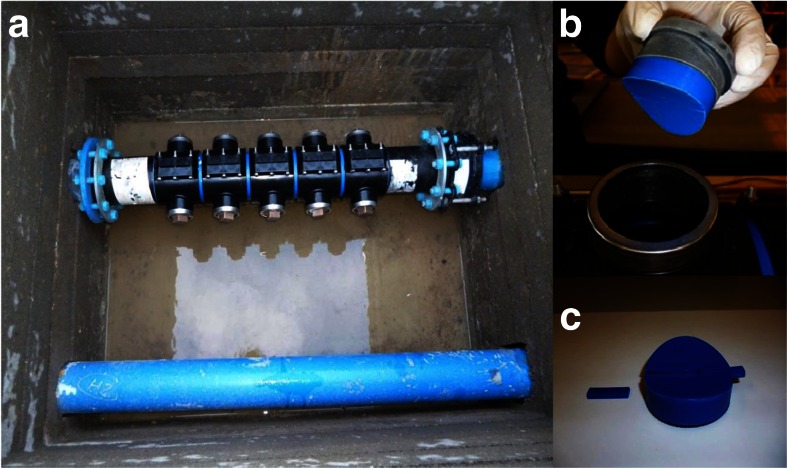
**a** Biofilm sampling device installed within a pipe section at one of the sampling sites. **b** Insertion of modified PWG coupons for in situ analysis of biofilms in the sampling device and coupon showing the curved profile designed to minimise disruption of boundary layer effects. **c** Coupon with two inserts designed for microscopy analysis and two identical outer areas for biofilm removal and subsequent DNA extraction

In order to characterise the community structure of biofilms under fully representative operational conditions, biofilm sampling devices (Fig. 1) designed by the University of Sheffield (UK) and containing modified Pennine Water Group (PWG) coupons (Deines et al. 2010) were installed at two networks in the south-west of the UK. The original smaller PWG coupons were adapted to have two larger areas in the coupon for nucleic acid extraction and two inserts (instead of one) for microscopy analysis, to maximise the limited space available (1 m longitude) for installing the biofilm sampling devices in the existent networks.

Sites were selected to be similar in all their characteristics (i.e. pipe diameter, material, etc.) excluding source water. However, due to factors outside the researcher’s control, the two systems were operated with different flow regimes during the studied period (Fig. S1 Supplementary Material). The surface water site is supplied with water from local springs and river abstraction. The water is treated by coagulation with aluminium sulphate to entrap particulate material, flocculation and removal of floc particles by dissolved air flotation. After this, the water is filtered using sand filters and granular activated carbon is used to absorb and remove organics. Chlorine is used for disinfection and as a residual. The groundwater site is a standalone borehole site, supplied with a mixture of water from ten boreholes. Treatment on site includes marginal chlorination using sodium hypochlorite solution for disinfection residual.

### Coupons and collection of biofilms

Two sampling devices containing modified Pennine Water Group (PWG) coupons were installed at the studied operational systems (Fig. 1a). The devices were made of high-density polyethylene (HDPE) and are 1 m long of 125-mm standard dimension ratio (SDR) 11. The coupons are curved to follow pipe curvature and thus minimise distortion of boundary layer effect including shear stress, turbulence regime and nutrient gradient. The coupons have a total area of 16.61 cm^2^ and two flat inserts of 1.15 cm^2^ dividing the outer section in two similar areas (Fig. 1c). The flat inserts allow for the study of biofilms, without disruption, using microscopy techniques. After the installation of the devices, sterile coupons were inserted at each site and biofilms were allowed to develop for 3 months, after this time, three coupons (two sampling sections each) were collected at each site.

### Biological and physico-chemical analysis of bulk water samples

On the date of coupon collection, samples (duplicates) from the water that supplied the systems were collected using designated containers for physico-chemical and conventional microbiological analysis via sampling taps located immediately upstream of the devices. Temperature and pH were measured in situ using a Hanna portable meter and probe HI 991003. All the other parameters (see Table 1) were obtained by later analysis of discrete water samples by an UK-accredited drinking water laboratory. Flow was measured by magnetic flow meters upstream of the coupon devices. For heterotrophic plate count (HPC) analysis, bacterial cultures were incubated at 37 °C for 48 h (2-day colony) and 22 °C for 72 h (3-day colony) and colonies counted after that period of time, following UK Standard Methods.

**Table 1:** Biological and physico-chemical parameters analysed in the bulk water supplying each biofilm sampling device (average ± standard deviation, *n* = 2)

	Surface water supplied site	Ground water supplied site
Colonies 2D 37 °C (No/ml)	0	0.5 ± 0.5
Colonies 3D 22 °C (No/ml)	5 ± 1.00	1 ± 1.00
E coli/100 ml	0	0
*Flow cytometer counts (cells/ml)	12.9 · 10^3^ ± 1540	7.1 · 10^3^ ± 599
Temperature (°C)	12.5 ± 0.08	9.2 ± 0.08
Turbidity (NTU)	<0.1	0.1 ± 0
Conductivity 20 °C (μS/cm)	298 ± 0	449 ± 6.01
Conductivity 25 °C (μS/cm)	330 ± 0	496 ± 6.5
pH	7.8 ± 0	7.6 ± 0
Alkal MO (mg CaCO3/l)	66.5 ± 0.5	202 ± 2.00
Ammonia as N (mg N/l)	<0.02	0.02 ± 0
Tot oxid N (mg N/l)	1	8.6 ± 0
Nitrite as N (mg N/l)	<0.003	0.003 ± 0
Nitrate as N (mg N/l)	1.009 ± 0.04	0.02 ± 0
Ammonia (mg NH3/l)	<0.01	0.01 ± 0
Nitrite (mg NO2/l)	<0.01	0.01 ± 0
Nitrate (mg NO3/l)	4.46 ± 0.04	37.95 ± 0.05
Orthophosphate (mg P/l)	<0.03	0.03 ± 0
Sulphate (mg SO4/l)	62 ± 0	18 ± 0
Chloride (mg Cl/l)	21 ± 0	18 ± 0
Silica (mg Si/l)	4.5 ± 0	3.7 ± 0
Free Cl2 (mg Cl/l)	0.3 ± 0	0.22 ± 0
Total organic carbon (mgC/l)	1.1 ± 0	<0.05
Al (mg/l)	<0.01	<0.01
Mn (mg/l)	0.002 ± 0	<0.001
Fe (mg/l)	0.01 ± 0	<0.01
Cu (mg/l)	<0.01	<0.01
Zn (mg/l)	<0.01	<0.01

*For flow cytometer, *n* = 5

Flow cytometry was used to estimate the microbial load entering the networks under ordinary operating conditions. Water was collected in sterile 50-ml tubes and transported in the dark and at 4 °C until analysed within 24 h of collection. SYBR® Green I (Molecular Probes, Invitrogen UK), used for staining nuclear double-stranded DNA, was diluted 1:100 with filtered dimethyl sulfoxide. 10 μl of SYBR® Green I 100× was added to 990 μl of the sample; the mix was vortexed and incubated for 15 min in the dark until measurements were carried out. The analysis was performed using a BD^TM^ LSR II Flow Cytometer System (BD Biosciences, UK). The samples were excited by a blue 488-nm laser and SYBR® Green I was detected by a 505-nm long-pass and a 530/30 nm band-pass filter set. Data were processed and analysed using the BD FACSDiva™ software (BD Biosciences, UK).

### Microscopy analysis

*Confocal laser scanning microscopy (CLSM)* was used to quantify the biofilm coverage on the coupons. Three coupons were studied for each site. Each coupon had two inserts, one of these inserts was used for SEM; hence, five inserts per sampling site were available for analysis using CLSM. The flat insert section was separated from the coupon and fixed in 5 % formaldehyde for 24 h and then transferred to phosphate buffer solution (PBS) and stored at 4 °C until analysed. After fixing, the inserts were stained with 20 μmol l^−1^ Syto® 63 (Molecular Probes, Invitrogen, UK) for 30 min at room temperature. Syto® 63 is a cell-permeative nucleic acid stain used to visualise cells (McSwain et al. 2005). Imaging was performed using a Zeiss LSM 510 Meta Confocal Florescent Microscope, and the LSM 510 Image Examiner Software was used to visualise the images (Zeiss, UK). Each insert was imaged for seven random fields of view, and the images were then processed to obtain a relative quantification of the biofilm at each layer as previously described (Fish et al. 2015).

*Scanning electron microscopy (SEM)* was used to visualise the appearance and coverage of biofilm developed on the coupons inserts. Inserts were fixed overnight with 5 % formaldehyde to preserve them until further analysis. Following this, inserts were fixed in 2 % aqueous osmium tetroxide for 1 h at room temperature. A series of ethanol dilutions in distilled water were used for dehydrating the inserts in 15-min steps as follows: 75 %, 95 %, two steps of 100 % ethanol, and 100 % over anhydrous copper sulphate. The inserts were immersed in a 50/50 % (*v*/*v*) solution of absolute ethanol and hexamethyldisilazane for 30 min and then transferred to 100 % hexamethyldisilazane for a further 30 min. Samples were air-dried overnight and then coated with 25 nm of gold using S150B sputter coater (Edwards, UK). Images were obtained with a Philips XL-20 SEM (Philips, Cambridge, UK) at an accelerating voltage of 20 kV.

### Biofilm removal and DNA extraction

To extract biofilm DNA from the coupons, first the two symmetric outer areas of each coupon were brushed to remove biofilm following the procedure used by Deines et al. (2010). After brushing, biofilm suspensions (two per each coupon) were concentrated in membrane filters as previously explained (Douterelo et al. 2013). Bulk water samples (three replicates of 1 l per site) were also filtered through 0.22-μm nitrocellulose membrane filters (Millipore, Corp.) for subsequent DNA analysis. Biofilm and bulk water samples were then preserved in the dark at −80 °C until DNA was extracted. To extract DNA, a method based on proteinase K digestion followed by a standard phenol/chloroform/isoamyl alcohol extraction was used (Neufeld et al. 2007).

### Pyrosequencing analysis

Bacterial 16S rRNA gene and fungal SSU gene tag-encoded pyrosequencing analysis was performed by Research and Testing Laboratory (Lubbock, TX, US) using the primers Gray28F (5′-GAGTTTGATCNTGGCTCAG -3′) and Gray519r (5′-GTNTTACNGCGGCKGCTG-3′) (Callaway et al. 2010) for bacteria and the 18S ribosomal small subunit primers F545 (3′-TGGAGGGCAAGTCTGGTG-5′) and Rev1021 (3′-TCGGCATAGTTTATGGTTAAG-5′) for fungi. Sequencing reactions were as described in Douterelo et al. (2013). utilising a Roche 454 FLX instrument (Roche, IN, US) with titanium reagents. In total, 93,519 16S rRNA gene sequences and 89,214 SSU-rRNA (18S) gene sequences were obtained from the biofilm and water samples analysed. Initially, sequences were cleaned by removing tags, primers and low-quality sequence ends. Chimeric sequences were detected using Black Box Chimera Check software (B2C2) (Gontcharova et al. 2010) and excluded from further analysis. Subsequently, sequences were denoised, assembled into clusters and queried using a distributed BLASTn.NET algorithm (Dowd et al. 2005) against databases derived from the NCBI. The BLASTn outputs were compiled and validated using taxonomic distance methods as previously described (Dowd et al. 2008).

To estimate microbial richness and diversity, Chao1 richness estimator (Chao, 1984) and Shannon diversity index (Shannon and Weaver, 1963). the software Quantitative Insights into Microbial Ecology (QIIME) (Caporaso et al. 2010) was used. Sequences were quality filtered, aligned and clustered using QIIME pre-established parameters and community analysis pipeline. Good-quality sequences were clustered into operational taxonomic units (OTUs) based on 97 % sequence similarity with the Uclust algorithm (Edgar, 2010). Then, representative OTUs were selected based on the most abundant sequences in the samples (Bik et al. 2012). Differences in bacterial and fungal community structure between samples were assessed using the relative sequence abundance at 97 % sequence similarity cutoff. The data was transformed by square root calculations, and Bray–Curtis similarity matrixes were generated using Primer-E v6 (PRIMER-E, Plymouth, UK) and visualised using non-metric multi-dimensional scaling (MDS) diagrams. Analysis of similarity statistics (ANOSIM) was calculated using the same Bray–Curtis distance matrix to test the significance of differences between samples. Sequencing data were deposited in the National Centre for Biotechnology Information (NCBI), Sequence Read Archive SRA 243897.

## Results

### Characteristics of the water supplied to the biofilm sampling devices

Due to operational constraints and circumstances beyond the author’s control, the flow in the groundwater site was reduced during the biofilm developmental period (see Fig. S1, Supporting Information). Results from the physico-chemical analysis and conventional microbial indicators can be seen in Table 1. Very low HPC and no coliforms (*Escherichia coli* per milliliter) were detected at either site. Flow cytometer counts were higher for the surface than the groundwater samples.

Conductivity, alkalinity and nitrate levels were higher in the groundwater samples compared to the surface water ones. Temperature, sulphate, chloride and total organic carbon (TOC) were higher in the surface water samples. On the other hand, pH (7.6 to 7.8) levels of ammonia (≤0.01 mgNH3/l), nitrite (≤0.01 mg NO2/l) and orthophosphate (≤0.03 mg P/l) were similar at both sites. Very low levels of metals (≤0.01 mg/l) and turbidity (≤0.1 NTU) were also measured at both sites. Free chlorine concentrations were slightly higher at the groundwater (0.3 mg/l) than at the surface water site (0.22 mg/l).

### Microscopic characterisation of biofilms

**Fig. 2:**
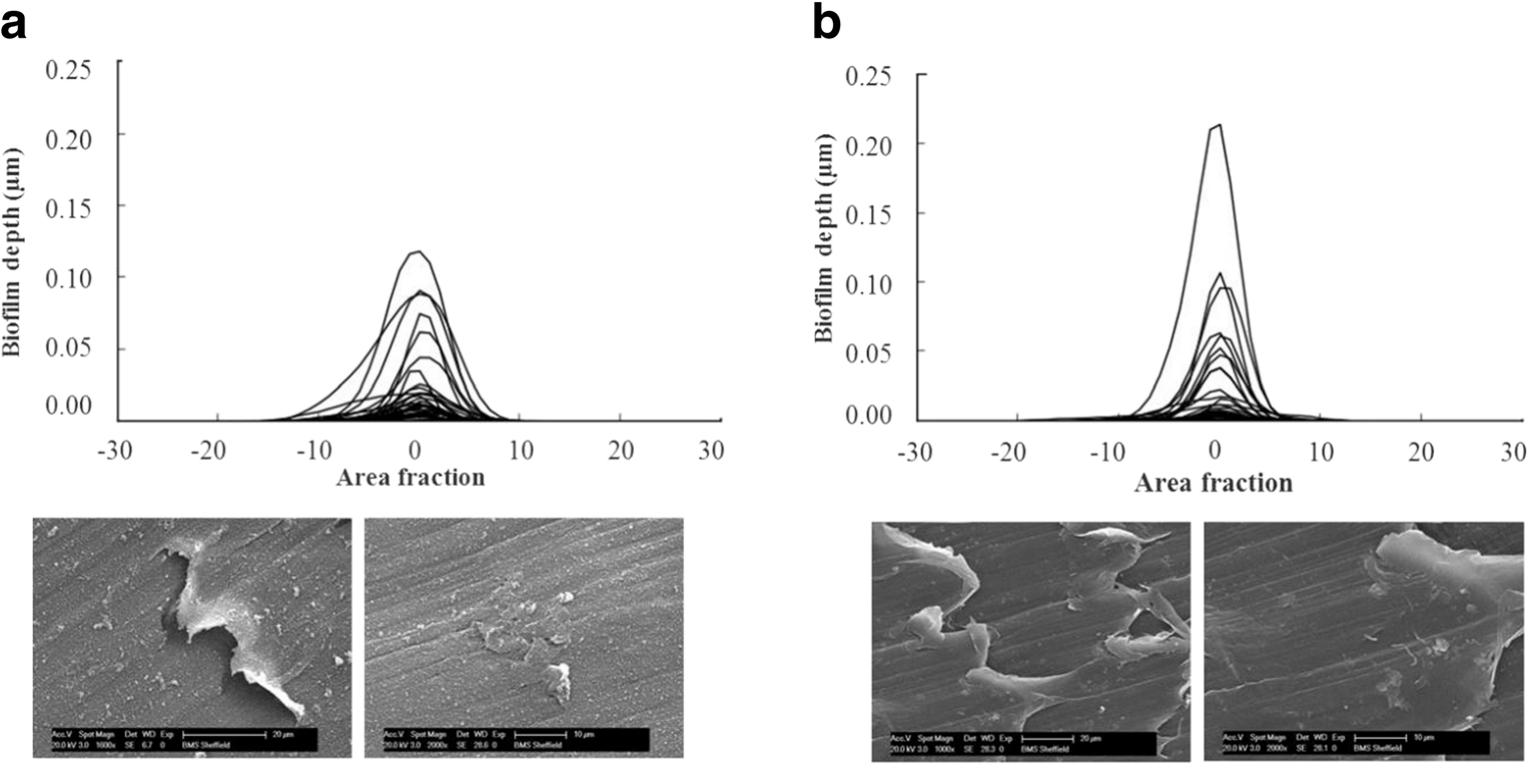
*Top*: Area distribution plots showing cell coverage on biofilms estimated by CLSM analysis. *Bottom*: SEM micrographs of biofilm grown on modified PWG coupons: **a** surface water and **b** groundwater

The microscopy analysis, CLSM and SEM, is shown in Fig. 2. The analysis of the CLSM images shows area distribution plots with the area coverage plotted against depth (aligned to the maximum area coverage at zero depth). Despite evident visual differences in biofilm coverage between sites, the analysis of the Z-stack images did not show statistically significant differences in the volumes of biofilm, since the average of the area fraction for both sites was similar and ranged from 0.14 to 0.15.

The SEM micrographs show that biofilms were not uniformly distributed on the surface of the coupons. Different layers of material were covering the inserts from each site, indicating that the deposition of material on the coupons was different between sites. The micrographs from the surface water (Fig. 2a) show an irregular rough surface with high concentration of granular material covering the insert. The groundwater supplied insert (Fig. 2b) shows a smoother surface, with fewer particles and a large even biofilm accumulation where several rod-shaped bacteria were observed.

### Bacterial–fungal community structure at sampling sites

**Fig. 3:**
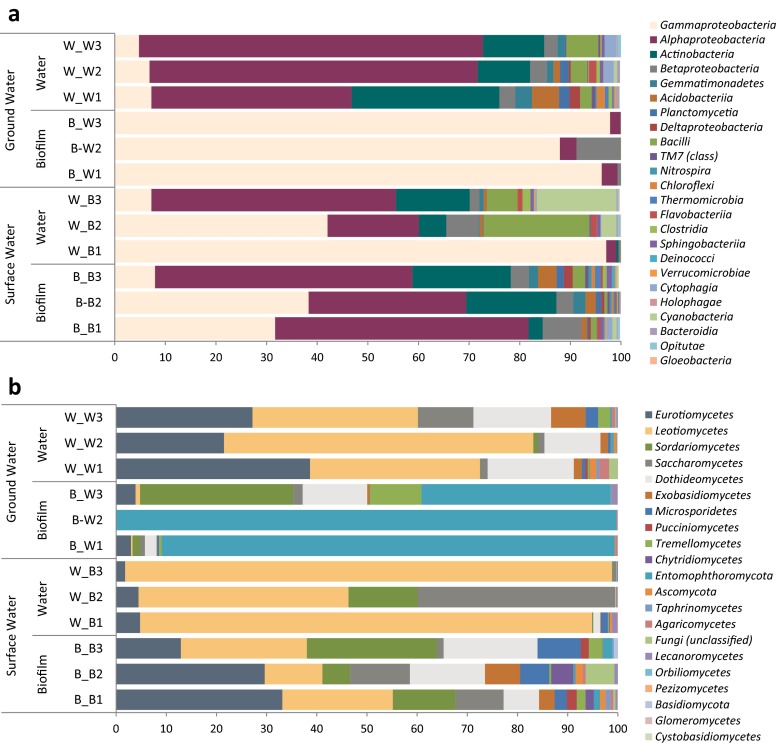
Relative abundance at class level of **a** bacteria and **b** fungi in biofilm and bulk water samples

**Fig. 4:**
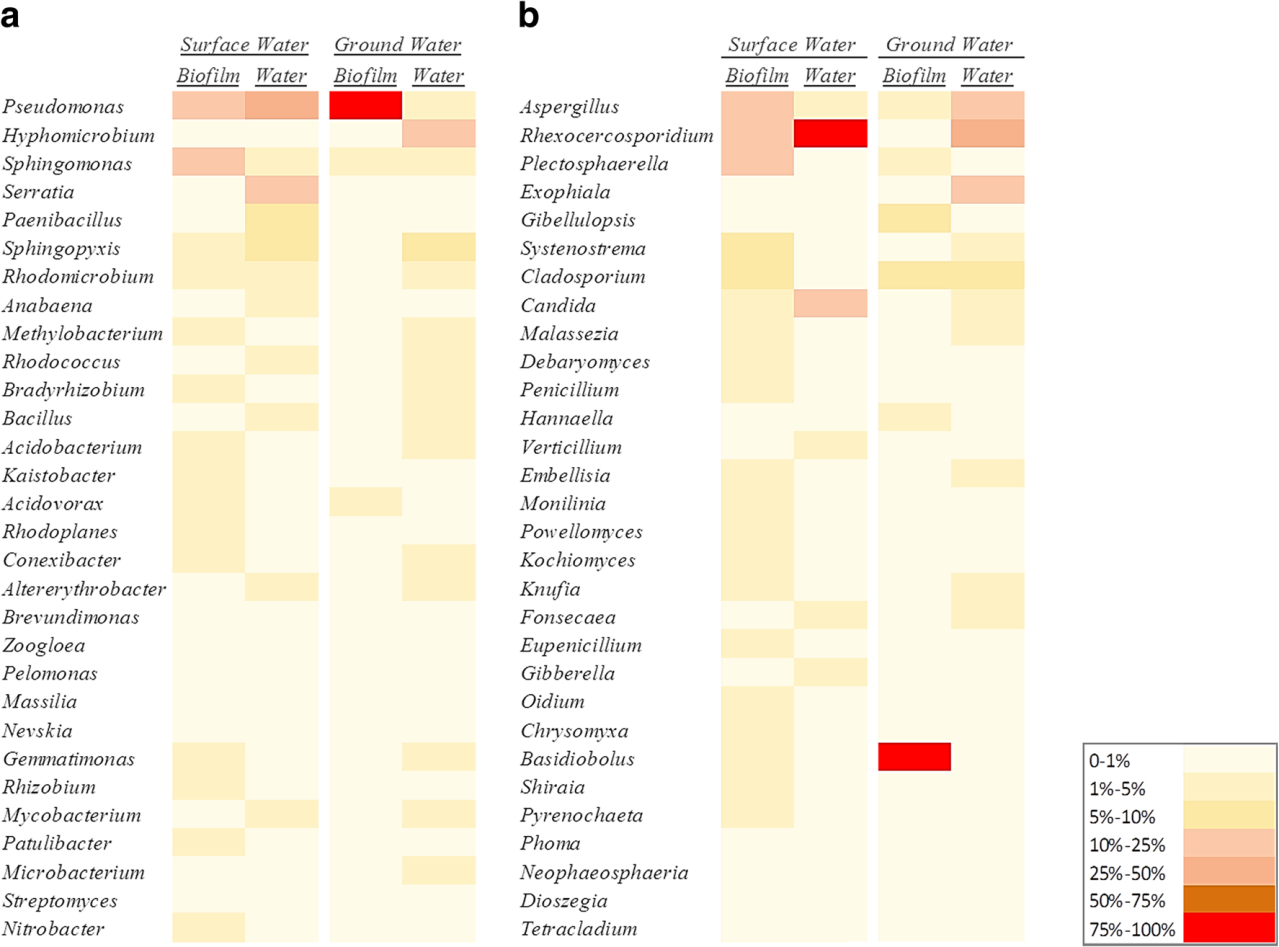
Heat maps showing the percentages of the most abundant bacteria and fungi at genus level within bulk water and biofilm samples (*n* = 3). **a** Bacteria and **b** fungi

Clear differences in the structure of the bacterial community at different taxonomic levels were found between sites. At class level (Fig. 3a), biofilm samples supplied with surface water presented high relative abundance of *Alphaproteobacteria*, *Gammaproteobacteria* and *Actinobacteria*. These bacterial groups were also abundant in the surface bulk water samples followed by *Cyanobacteria* and *Bacilli* in lower percentages. The bacterial communities from bulk water samples from the groundwater site were dominated by *Alphaproteobacteria*, *Actinobacteria* and *Gammaproteobacteria*. However, the biofilm samples from this site, with very low flow through, were mainly composed of *Gammaproteobacteria* (>88 %). At genus level (Fig. 4a), *Pseudomonas* was consistently found in all the samples, but it was particularly abundant in biofilm groundwater samples (>87 %). Another genus present in planktonic and biofilm samples was *Sphingomonas* (5–25 %). *Sphingopyxis* was abundant in the bulk water samples of both sites representing 5–10 % of the total bacterial community. In surface water samples, *Serratia* was an important member of the bacterial community but *Hypomicrobium* dominated in ground bulk water samples.

Differences in the structure of the fungal community were also found between the two sites and planktonic and biofilm phases. At class level (Fig. 3b), *Eurotiomycetes* and *Leotiomycetes* were the main constituents of the fungal community, abundant in the bulk water at both sites. *Leotiomycetes * was the main class (41–97 %) represented in the surface water biofilm samples. *Entomophthoromycota* strongly dominated the community composition (37–99 %) of biofilm samples supplied with groundwater. At genus level (Fig. 4b), species belonging to *Aspergillus*, *Rhexocercosporidium* and *Plectosphaerella* were represented in the fungal community from all the samples. However, several genera were primarily found in bulk water samples including *Candida* and *Fonsecae* while other genera were abundant in biofilms such as *Plectosphaerella* and *Cladosporium. Basiodobolus* was the main fungal genus (>75 %) in all the groundwater biofilm samples.

Non-metric MDS plots of the relative abundance of bacteria and fungi at 97 % sequence similarity cutoff (Fig S2, Supporting Information) showed significant differences between samples sites regarding the source of water supplied and between the planktonic and attached phases with the surface water supplied biofilm the most different (*p* < 0.05).

### Rarefaction: diversity and richness estimations

**Fig. 5:**
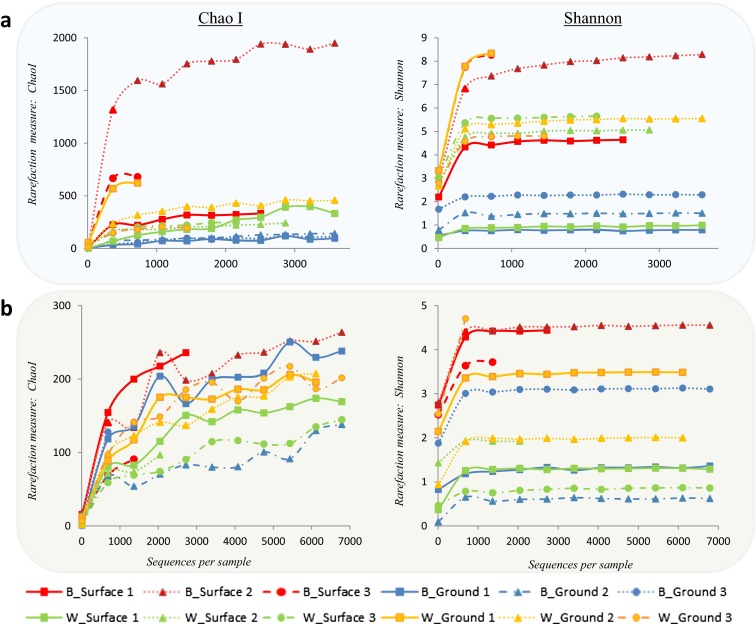
Rarefaction curves at 97 % of sequence similarity for water and biofilm samples. Rarefaction curves were obtained for Chao1 index richness estimator and Shannon diversity estimator. **a** Bacteria; **b** fungi. In the legend: *B* = biofilm, *W* = water, and the *numbers* indicate the biological replicate

Figure 5 shows the results for the estimations of diversity and richness for bacterial (a) and fungi (b) for planktonic and attached communities from the ground and surface water supplied sites. Higher diversity and richness were estimated for bacteria when compared with fungi for most of the samples with the exception of groundwater biofilm samples which showed low diversity and richness. The rarefaction curves showed that, despite high variability between some of the biological replicates, the general trend was for the biofilm samples supplied with surface water and a higher flow rate to have higher microbial diversity and richness. The samples with least bacterial diversity and richness were those obtained from biofilms supplied with groundwater and with a continuous low flow rate through the pipe. Bulk water samples from the groundwater site had similar or higher bacterial diversity and richness when compared with surface water samples. For fungi, the groundwater bulk water samples presented, in average, higher richness and diversity when compared with the surface water samples.

## Discussion

### Biofilm development under operational conditions

Several factors measured in bulk water samples including total cell concentration, TOC and temperature were higher in the surface water site when compared with the groundwater site. Despite these factors that suggest greater potential for biological growth and biofilm formation in the surface water site, the cell coverage of biofilms calculated from the CLSM images did not show significant statistical differences between the sites. Similarly, the analysis of the sequencing data showed a general trend for bulk groundwater samples to have higher bacterial and fungi diversity when compared with surface water samples, indicating that the groundwater contained as many microorganisms as the surface water despite common understanding that groundwater typically promotes lower microbial occurrence due to removal and filtering processes while travelling through soil and permeable rock (Abu-Ashour et al. 1994).

Regardless of higher microbial diversity being detected in most of the bulk groundwater samples when compared with surface water, biofilms developed under groundwater supply showed very low diversity which can be explained by the flow rates maintained in the network. In a previous study, where pyrosequencing was used to study the effect of hydraulic regimes on biofilm development in an experimental DWDS, highly diverse biofilms were observed under varied flow hydraulic regimes (Douterelo et al. 2013). In agreement with this study, Holinger et al. (2014) suggested that operational parameters and the way the water is distributed have a greater influence on the microbial communities present in final tap water than geographical location, land use and the source water. Similarly, Sly et al. (1988) observed that water velocity significantly influenced the development of biofilms in drinking water pipes. Furthermore, van der Wielen et al. (2013) suggested that the type of source water used for drinking water production had a limited influence on the occurrence of some opportunistic pathogens found in unchlorinated drinking water systems. This study shows that water source provides the “seed” for biofilm development, but not all microorganisms traveling in the water are successful inhabitants of attached communities, and that hydraulic regimes play a central role in shaping biofilm physical and community structure. It should be noted that other factors such as nutrient supply will also have an impact.

The groundwater biofilm community was dominated by a limited number of microbial genera, the bacteria *Pseudomonas* and the fungi *Basidiobolu*s (Fig. 4). *Pseudomonas* has been found to be a ubiquitous bacterium involved in the process of biofilm formation and highly abundant under low varied flow conditions (Douterelo et al. 2013). *Pseudomonas* is also a denitrifying bacterium and since denitrification is favoured in areas of stagnant water (Nawrocki et al. 2010) it is reasonable that it is dominant in the groundwater site located in an agricultural area with high levels of nitrates. Despite of the key role of *Pseudomonas* in the formation of biofilms in DWDS, there are no regulations restricting the presence of these microorganisms in the water; however, they can be good indicators of biofilm risk development in water networks.

The diverse microbial community found in the bulk water samples at the groundwater site confirms the restricted vision we previously had of groundwater supplied systems which is probably a result of the limited number of groundwater studies conducted in these environments and the type of analytical techniques, mainly culture-dependent, used to characterise them.

### Bacterial community structure (plankton and biofilm)

*Proteobacteria* and within this group *Gammaproteobacteria* were consistently highly abundant in all the samples with the exception of groundwater bulk samples where *Alphaproteobacteria* were predominant. *Proteobacteria* have been found in different drinking-water-related ecosystems (Williams et al. 2004; Lee et al. 2005; Eichler et al. 2006; Poitelon et al. 2009; Navarro-Noya et al. 2013), and this study confirms the ubiquity of this taxonomic phylum in operational DWDS supplied with different water sources and under different hydraulic regimes. Several bacteria in this study were common inhabitants of biofilms (e.g. *Pseudomonas*, *Sphingomonas* and *Acidovorax*) while others such as *Rhodococcus*, *Bacillus*, *Alterythrobacter* and *Mycobacterium* were mainly abundant only in water, confirming previous observations that not all the microorganisms transported in the water are successful in forming biofilms (Douterelo et al. 2013). This result shows the limitations of current monitoring strategies where the quality of drinking water is measured using bulk water samples and culture methods.

*Hypomicrobium* was the main microorganism found in groundwater bulk samples and has previously been found in the biomass obtained from flushing cast iron pipes in a chlorinated DWDS (Douterelo et al*.*2014b) and in a chloraminated distribution system simulator (Williams et al. 2004). The ability of *Hypomicrobium* to deposit iron and manganese on pipes makes the presence of this organism problematic, since it has been associated with water discolouration problems (Sly et al*.*^1988^) and discolouration is a major issue that water supply companies are facing in the UK and internationally.

The differences in community structure between the sites observed here in combination with the author’s previous data on biofilms developed under different hydraulic regimes but with the same water (Douterelo et al. 2013; Douterelo et al. 2014c) suggest that while source water influences the biofilm composition and structure, the hydraulic regimes have a central role in shaping biofilm development. However, to corroborate these preliminary results, further experimental data from both sites under the same hydraulic regime would be needed. Hence, the manipulation of hydraulic condition is potentially a readily applicable method to control biofilms and should be considered when managing DWDS. Furthermore, these results show that current monitoring strategies used to monitor drinking water quality based on analysing bulk water samples with culture-based techniques are ineffective since they do not take into account biofilm-associated risks. This study suggests that other microorganisms such as *Pseudomonas* and *Hypomicrobium* have the potential to be used as indicators of key process in drinking water supply systems including biofilm formation and discolouration.

### Fungal community structure (plankton and biofilm)

There is limited published research on fungi in DWDS, and what is available is based on information gathered from bulk water samples from taps using culture-based methods, hence providing a limited understanding of the real diversity of fungi in distribution systems. Taking into account these limitations, previous research found that fungi are more likely to be found in systems supplied with surface water than groundwater (Hageskal et al. 2006; Hageskal et al. 2007; Pereira et al. 2009). However, the water samples analysed here showed greater fungal diversity in most of the groundwater samples when compared to the surface samples. This result suggests that despite previous observations, groundwater is an important source of fungi in DWDS, favouring the development of multi-species biofilms in combination with bacteria. Several genera found in this study including *Aspergillus*, *Penicillium* and *Cladosporium* have been isolated using culture-dependant methods in drinking water systems such as water treatment plants (Sammon et al*.*^2010^) or hospital water (Hayette et al. 2010). The common detection of these fungi in previous drinking water studies is probably associated with the selective characteristics of the culture techniques used to identify them. Here, using culture independent methods, we have detected other fungal genera not normally reported in DWDS. As an example, *Rhexocercosporidium* was highly abundant in the surface water samples, this is a phytopathogenic fungus commonly found in soils and associated with farming systems (Kohlmeier et al. 2005). The area where the sampling device was located is dedicated to agriculture so the possibility of contamination of the source by fungal spores is relatively high. *Rhexocercosporidium* has been previously reported in drinking water samples supplied with groundwater in the Netherlands (van der Wielen et al. 2013). but no other references to this fungus within biofilms in DWDS were found. Similarly, fungi detected here such as *Penicillum*, *Gibellulopsis*, *Gibberella* and *Phoma* are usually found in soils; these can enter as spores but also as vegetative cells through leaks, when events of low or negative pressure occur (Collins and Boxall, 2012) or during maintenance of the system (Doggett, 2000). Fungi detected in this study such as *Exophiala* have been classified by Göttlich et al. (2002) as common inhabitants of DWDS and others such as *Verticillium* and *Phoma* as transitory species, defining transitory species as those that might either enter the systems through breaks or/and originated from contamination (Sonigo et al. 2011). However, further research is needed to establish how and when these fungi might enter in the system and form part of biofilms and their potential use as indicators of DWDS contamination.

As observed for bacteria, several fungi were predominant only in biofilms (e.g. *Basidiobolus* and *Plectospherella*) showing that they have an enhanced capability to adhere to pipe surfaces when compared with other genera mainly present in water such as *Verticillium* and *Fonsecaea.* Clear differences in the fungal community structure between biofilms at both sites were also detected. *Gibellulopsis*, *Hannaella* and *Basidiobolus* were abundant in groundwater biofilm samples and *Systenostrema*, *Penicillium* and *Candida* in surface water biofilms. Although *Candida* is considered as a potential pathogen, it has been found in DWDS elsewhere (Doggett, 2000; Brinkman et al. 2003) and there is no evidence that their pathogenicity results from their presence in drinking water (Sonigo et al. 2011). The high diversity of fungi found in this study suggests that further consideration should be given to these microorganisms in drinking water research. The control of fungi n DWDS can be challenging as they can form spores which tend to aggregate with each other and with other particles increasing their resistance to disinfection (Mamane-Gravetz and Linden, 2005; Sonigo et al. 2011), and they can be more resistant to disinfection than bacteria (Pereira et al. 2013).

### Associations between bacteria and fungi in DWDS

This study shows that both surface and groundwater planktonic samples had a diverse fungal community, which plays an important role in the subsequent formation of biofilms in DWDS, ultimately shaped by the hydraulic regime in the system. It has been shown that fungi normally colonise pre-established bacterial biofilms and because they have different ecological requirements, it has been suggested that this might indicate a positive relationship between these two types of microorganisms (Doggett, 2000). As a specific example, the bacteria *Pseudomonas* and the fungi *Basidiobolus* were coexisting in the biofilms analysed here. *Basidiobolus* normally lives on decomposing organic matter and similarly to other fungi has extracellular enzymes that allow them to degrade high molecular weight compounds, releasing secondary metabolites which can be used by other microorganisms, in this case potentially *Pseudomonas.* According to Larson (1989). fungi can favour bacterial growth in DWDS by two different processes: (i) chlorine can react with fungi reducing the chlorine residual and indirectly favouring bacterial growth and (ii) fungal mycelia can generate a substrate favourable for bacterial attachment and growth. Experiments using laboratory models have also shown that fungal hyphae can facilitate bacterial mobilisation in the environment (Harms and Wick, 2006). facilitating the colonisation of new sites along the pipe for bacteria, but this hypothesis and the ecological interactions between them will need further research.

Bacterial–fungal interactions within mixed species biofilms influence biofilm pathogenic potential and stress resistance (Frey-Klett et al. 2011), thus affecting the performance of the drinking water infrastructure. The information obtained in this study is the first step towards understanding the complex interactions occurring in real DWDS and to establish useful parameters and methods to effectively monitor these systems and to guarantee the delivery of good-quality water.

## Electronic supplementary material

ESM 1 Supporting Information. Details on flow rates (Fig. S1) and the MDS analysis based on Bray-Curtis similarities of the relative sequence abundance (Fig. S2) are available in the supplementary material. (PDF 215 kb)
