# Supplementary material for: Microbial analysis of in situ biofilm formation in drinking water distribution systems: implications for monitoring and control of drinking water quality
Source: Appl Microbiol Biotechnol. 2015 Dec 5;100:3301–11. doi: 10.1007/s00253-015-7155-3 (PMC4786615; doi:10.1007/s00253-015-7155-3)
Supplement: Supplementary file 1 — Supporting Information. Details on flow rates (Fig. S1) and the MDS analysis based on Bray-Curtis similarities of the relative sequence abundance (Fig. S2) are available in the supplementary material. (PDF 215 kb) [file 253_2015_7155_MOESM1_ESM.pdf]

Applied Microbiology and Biotechnology

Microbial analysis of *in situ* biofilm formation in drinking water distribution systems: implications for monitoring and control of drinking water quality.

I. Douterelo[1] M. Jackson[2] C. Solomon[2] and J. Boxall[1]

1. Pennine Water Group, Department of Civil and Structural Engineering, Mappin Street, University of Sheffield, Sheffield, S1 3JD, UK.

2. Wessex Water, Claverton Down Rd, Bath, Somerset BA2 7WW, UK.

Correspondent author:

Isabel Douterelo

e-mail: i.douterelo@sheffield.ac.uk

Telephone: +44 (0) 1142 225767

Fax: +44 (0) 114 222 5700

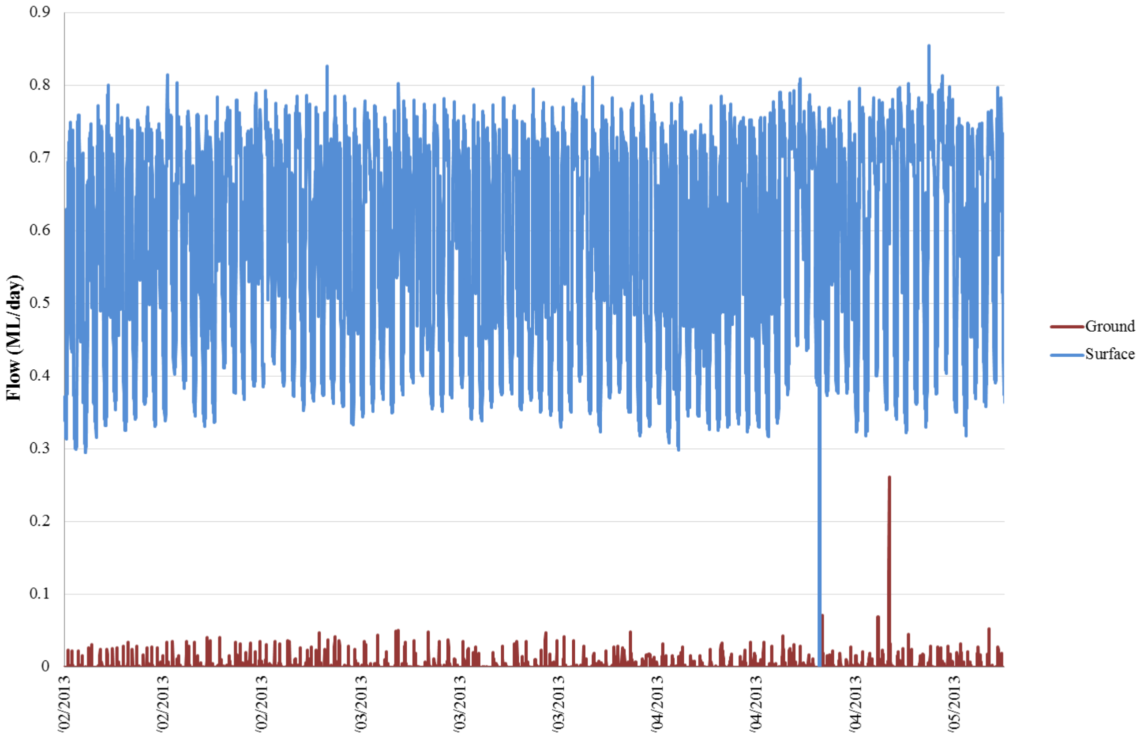

Figure S1: Flow rates through pipeline containing the two biofilm sampling devices during the 3 months that the experiment took place.

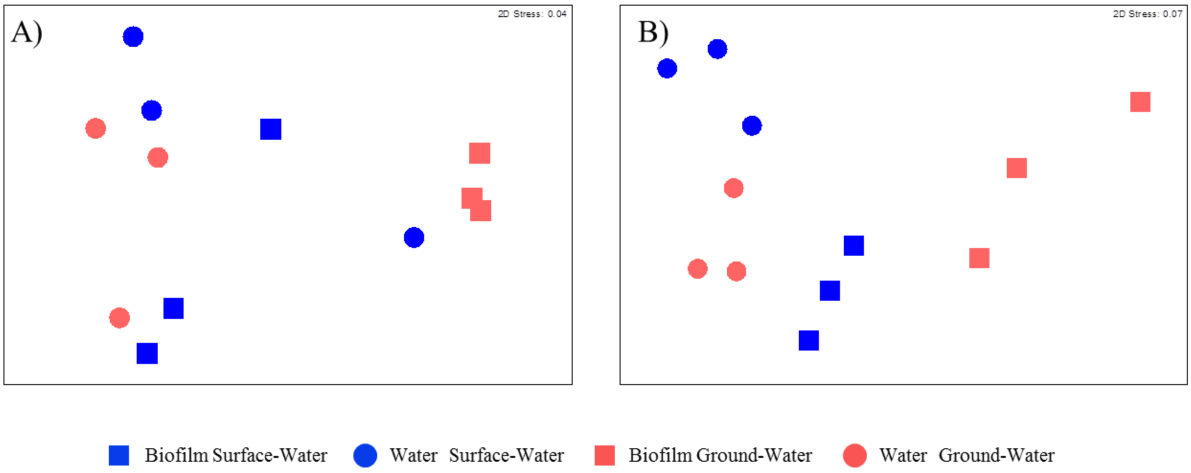

Figure S2: Two-dimensional plot of the Multi-Dimensional Scaling analysis based on Bray-Curtis similarities of the percentage sequence abundance at species level (A) bacteria and (B) fungi. Symbols represent individual samples and are coloured based on sample type.
